# Feasibility of engaging parents attending an adult weight management programme with child weight management support: a mixed methods study

**DOI:** 10.1136/bmjpo-2024-002975

**Published:** 2025-01-17

**Authors:** Ruth Mears, Aidan Searle, Deborah Sharp, Russell Jago, Julian PH Shield

**Affiliations:** 1 Centre for Academic Primary Care, Bristol Medical School, University of Bristol, Bristol, UK; 2 Population Health Sciences, Bristol Medical School, University of Bristol, Bristol, UK; 3 NIHR Bristol Biomedical Research Centre Diet and Physical Activity Theme, Translational Health Sciences, Bristol Medical School, University of Bristol, Bristol, UK

**Keywords:** Child Health, Obesity, Qualitative research

## Abstract

**Objective:**

To explore whether parents’ attendance at a commercial adult weight management programme (WMP) offers an opportunity to identify and signpost families to child weight management support, if appropriate to a child’s weight status.

**Design:**

Mixed methods study including a cross-sectional online survey and semistructured telephone interviews.

**Setting and participants:**

Parents attending Slimming World (UK-based adult commercial WMP), with one or more children aged 5–11 years, were invited to take part. There were 396 survey and 18 telephone interview participants.

**Results:**

Most parents (78%) attending the adult WMP and worried about their child’s weight were receptive to being offered support for their child. Nearly all (98%) of these parents were happy for the adult WMP to signpost to this support. Nearly half of parents (47%, n=122/262) not worried about their child’s weight were still interested in a child height and weight check. The preferred intervention format and delivery possibilities differed among parents, with ‘no-one size fits all’, while recognising that change takes time, and weekly sessions may be too frequent. Parents were clear that the focus of support should be on healthy lifestyle not weight. Many parents felt a parent ‘peer support’ group would be helpful.

**Conclusions:**

Parents actively addressing their own weight, through an adult WMP, are receptive to being offered and signposted to support for their child, where they have concerns about their child’s weight. These findings support a new referral pathway into child weight management services, through parents attending an adult WMP.

**WHAT IS ALREADY KNOWN ON THIS TOPIC**

**WHAT THIS STUDY ADDS**

- This study suggests most parents attending an adult weight management programme with concerns about their child’s weight are receptive to being signposted to support for their child.

**HOW THIS STUDY MIGHT AFFECT RESEARCH, PRACTICE OR POLICY**

- Our study supports the potential of a new referral pathway to child weight management programmes, through parents attending adult weight management programmes.

## Introduction

 Obesity in childhood increases the risk of cardiovascular and metabolic disorders in adulthood^1^,^4^; however, normalisation of weight before or during puberty reduces risk.^5^ Between 2019/20 and 2020/21, the National Child Measurement Programme (NCMP) reported the highest annual rise in obesity prevalence in children, since the programme began in 2006.^6^ This unprecedented increase was attributed to the impact of the COVID-19 pandemic.^6^ After the pandemic, prevalence figures from 2021/22 had improved slightly but remain worryingly high.^7^

In England, children living with overweight or obesity may be referred to lifestyle weight management programmes by healthcare professionals, including general practitioners.^8^ These programmes often experience difficulties in recruitment, engagement and retention of children and their families.^9^ Individual and family demands, lack of parental support or motivation, and lack of awareness of programmes are all barriers to engagement and retention within children’s weight management programmes.^8^

Adults with overweight or obesity may seek help to reach a healthier weight by joining Slimming World. Slimming World is a commercial weight management programme (WMP) with a membership base of around 700 000 and aims to improve weight through a healthy eating approach and personalised support to change both eating and physical activity behaviours (in-person group sessions or online).^10^ Parents living with obesity are more likely to have children affected by obesity.^11^

NICE guidelines state that referral to child lifestyle weight management programmes should take place when the individual and their family are ready and willing.^8^ The period when a parent is taking steps to address their own weight may represent an opportune moment to engage their children (if affected by overweight) in weight management support. The parent is motivated to make changes to their own lifestyle and even without any further intervention, there may be indirect benefits for the child.^12 13^

NICE guidance states that parents should be encouraged to ‘take main responsibility for lifestyle change in children who are living with overweight or obesity, especially if they are younger than 12 years’.^14^ The optimal role of parental involvement in adolescent obesity treatment is less clearly defined and complicated by the young person’s increased desire for autonomy and independence.^15^ For this reason, our study focuses on parents with primary school-aged children (5–11 years old).

This study examined whether parents of children aged 5–11 years old attending an adult WMP are: (1) receptive to their child’s height and weight being measured; (2) interested in receiving support for their child, if they are worried that their child is overweight, or their child is identified as overweight (through a height/weight check); and (3) willing to be signposted by the adult WMP to support for their child. Finally, our study also examined formats of support, parents would like to help their child reach a healthier weight.

## Methods

### Participants and recruitment

Parents attending Slimming World, with children aged 5–11 years, were eligible to take part in an online survey and telephone interview. The survey was offered to all parents, regardless of their perception of their child’s weight status. The telephone interviews focused on parents worried about their child’s weight.

Participants were recruited via an advert on the members-only section of the Slimming World website. The study was also highlighted in a weekly email communication to Slimming World group Consultants, asking them to share the survey link in their weekly group session with eligible members. Telephone interview participants were also recruited from survey respondents.

### Survey data collection and analysis

The survey was available via the ‘Online Surveys’ platform (https://www.onlinesurveys.ac.uk/). Free text responses to survey questions were analysed in N-vivo through content analysis. All other survey data were analysed in Excel and Stata v18.0 and reported using descriptive statistics. The index of multiple deprivation (IMD) decile was derived from the participant postcodes, to measure socioeconomic status for English residents.

### Telephone interview data collection and analysis

Telephone interviews were conducted by RM (a primary care research fellow) and facilitated by an interview topic guide developed by RM, RJ, DS and JHS. Interviews were audio-recorded, transcribed, and anonymised with data stored and managed in NVivo 1.6.1 software. Data were analysed by RM and AS (a qualitative researcher) following the process of Braun and Clarke.^16^ The analysts familiarised themselves with the data and noted initial ideas prior to ‘open coding’ the dataset. The analysts then met to reach consensus on a definitive coding frame. Themes were identified and mapped to survey findings to contextualise parental views of parent vs family-led interventions, methods of delivery and interest in parent-peer support groups.

### Patient and public involvement

Patients or the public were not involved in the design, or conduct, or reporting, or dissemination plans of our research.

## Results

There were 396 online survey participants (98% female) with a median parental Body Mass Index (BMI) of 34.5 kg/m^2^ (IQR 30.2 to 39.6), median age of children within relevant age group (5–11 years old) of 8 years (IQR 6 to 10) and median IMD decile for participants residing in England (n=338) of 5 (IQR 3 to 8). Data saturation was achieved after 18 telephone interviews: average interview length 43 min; median parental BMI of 37.8 kg/ms^2^ (IQR 32.0–40.0), child median age 9 years (IQR 7 to 11) and median IMD decile of 4 (IQR 3 to 6). The survey was conducted between August and October 2020, the telephone interviews between March and November 2021.

### Receptiveness to a child weight status check and support

**Figure 1:**
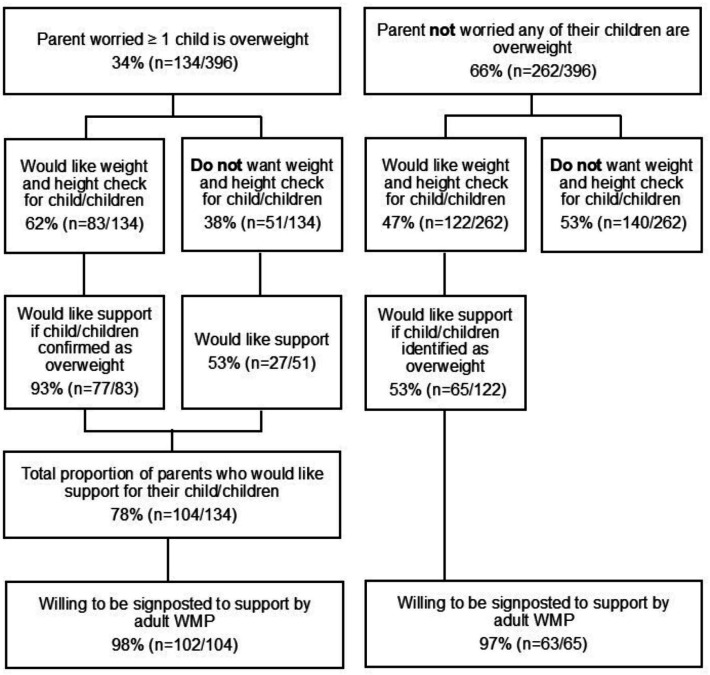
Parental interest in a child weight status check and receptiveness to being offered support (Source: online survey data).

Most parents (78%, n=104/134) worried about their child’s weight wanted support to help their child attain a healthier weight, and 98% (n=102/104) were willing to be signposted to this support by the adult WMP (figure 1). Nearly half (47%, n=122/262) who were not worried about their child’s weight were still interested in a height and weight check for their child, of whom 53% (n=65/122) indicated that they would be receptive to child weight management support if the check identified their child as having overweight or obesity (figure 1).

### Reasons for not wanting a weight status check

Just under half of parents (n=191/396, 48%) would not want their child’s weight status measured. Five main themes emerged from free text survey responses to explain this (table 1). Most parents would decline a weight and height check for their child because they felt they already knew their child’s weight status, or how to assess it. A minority of parents expressed concerns about weighing their child, and a few parents stated that checks were already conducted elsewhere.

**Table 1:** Parent’s reason for not wanting a child weight status check (Source: online survey, free text answers, n=191)

Theme	Quote
I already know my child is overweight	‘I know they are definitely overweight.’ (N158, Female 46–55 years)‘She is chunky I would appreciate help in managing her weight rather than simply being told she’s fat.’ (N166, Female 36–45 years)
I already know my child is a healthy weight	‘She is a healthy size, fit and active and fits in the clothing band easily.’ (N389, Female 26–35 years)‘I believe my child is at a healthy weight and if I became concerned about this, I would get it looked into.’ (N74, Female 26–35 years)
Checks already undertaken elsewhere	‘They are under paediatric care and gets check-ups anyway.’ (N11, Female 36–45 years)‘My son has asthma and is regularly monitored for height and weight’ (N67, Female 36–45 years)
Parent feels confident in how to assess the weight of their child	‘I am able to access with my eyes, the clothes they wear as to if they are of normal weight for their height.’ (N41, Female 36–45 years)‘I would notice if my child looked overweight after struggling with my own weight.’ (N117, Female 26–35 years)‘I am happy with my assessment as a parent, using the growth centiles.’ (N28, Female 26–35 years)
Parental concern about weighing their child	‘I help my daughter without her direct knowledge as I don’t want her to think she has a weight issue in case it affects her mentally.’ (N168, Female 46–55 years)‘I think it’s more about how they look rather than a number on a scale and I think weighing a child would be a detriment to them and cause them to worry about something that they shouldn’t at that age.’ (N89, Female 26–35 years)

### Intervention design: parent vs family-based interventions

Online survey data identified preference for a family-based intervention (55%) compared with a parent-led intervention (45%). The telephone interviews explored this further (table 2). Some perceived parent-led interventions to be useful as they believed parents are effective agents of change, without making weight an ‘issue’ for the child. Others recognised the benefit of an external person talking directly to their child through a family-based intervention.

**Table 2:** Parent-led vs family-based intervention (Source: telephone interview data n=18)

Topic	Theme	Quotes
Parent-led	Parents can be effective agents of change	‘Ultimately, it’s me that’s providing him with his meal and it’s me that’s saying yes or no, you know, I’m very much in control of what goes in that boy’s mouth and what that boy does with his body, so that way then there isn’t really an awful lot of point in (child) being there.’ Interview 11
	Avoids weight becoming ‘an issue’	‘So I don’t want her to think that even her own family thinks that she’s got an issue. She gets that at school, she doesn’t need anyone else.’ Interview 7
Family-based	Benefits of external person talking directly to child	‘You can stand on your head trying to tell them how to do long division, they’re never going to take it in as quickly or as easily or with as much faith as they will from a maths teacher. Maybe it’s just something which is innate in kids that they need to learn from other people as well as their parents… so having that external person involved, I can really see that that would be beneficial.’ Interview 2‘It would need to be somebody else talking to my daughter because she wouldn’t take it from me.’ Interview 10‘I think that works best because they’re getting an understanding of what needs to be changed.’ Interview 18
	Child needs to understand healthy choices	‘We’ve tried to do it by myself and its obviously not working for whatever reason, so if I attended a parent only intervention and I came back and put things in place for (son) that I could do, but once he’s at his dad’s and once he’s at school my steer of influence would stop and he’d be free to do whatever he wants.’ Interview 3

### Intervention design: method of delivery

Online survey data suggested the majority preferred a face-to-face intervention (64%), followed by mobile phone app (53%), web-based (50%), written information pack (4%) and other (1%).

The telephone interviews identified a range of views about the benefits and issues with different methods of delivery: the emerging theme being ‘no one size fits all’ (table 3). Some preferred a ‘more personal’ face-to-face contact, allowing for a human connection to be established. Others felt that a virtual group may be easier with less travelling and the child feeling more confident in a virtual setting. The downside of virtual groups included discomfort in not knowing the people on the call, and that the child may be easily distracted within their own home.

**Table 3:** Intervention format and delivery (Source: telephone interview data, n=18)

Topic	Theme	Quotes
Method of delivery	No-one size fits all	‘I think it can vary hugely with the participants because there’s something good about going to a place and meeting other people face to face, and in reality, however for a lot of people who are ending up at these groups they might feel much more confident doing it virtually than physically walking through the door and being stood there in front of other people.’ Interview 1‘There could be some quite generic information on the mobile phone app and then I guess the more personalised bit would be finding out from the child what they, or the parent, what support they wanted and addressing any queries.’ Interview 10
Frequency of support	Change takes time	‘I just think people just need to be given a bit of time to kind of register the changes that need to happen and then put those changes into place and then keep going with it for a minute before you’re back again the following… No, I think weekly’s just not long enough really.’ Interview 11
Staffing of intervention	Benefits of a multiprofessional approach	‘I would welcome a psychologist who can talk about emotional eating, I’d welcome a dietitian who can tell children and their parents… just educate people about food.’ Interview 3
	Need for trained and experienced staff	‘As long as they’ve had the training and we need to understand that it is not just about the food we eat, it’s about the way we feel about the food we eat as well, I think then as long as they’ve been trained in that respect I don’t think it really matters who gives that information.’ Interview 4
Acceptability of weight monitoring	Weight monitoring acceptable, if emphasis to child is on health, not weight	‘Coming back to what I said to her, look it doesn’t matter what it says on the scale, it is about you being healthier and I don’t want you to think that you’ve got to now go starving yourself and stuff like that. I think it’s all about how that’s put to the child.’ Interview 4‘What I wouldn’t be happy with and what has completely mucked with my mind is for him to be told that it’s a fantastic thing that you’ve lost this weight and told the figures and stuff.’ Interview 11‘And weight isn’t everything, right? It’s much more now, you know, being able to move and run around like children and just being lively and healthy.’ Interview 7
	Importance of privacy of weight measurements	‘From (son 2)’s point of view I think he’d prefer that nobody knew about it, so I think he’d prefer it was quite discreet.’ Interview 3‘Just don’t want her to feel different or stand out in front of people as… the kids start to know don’t they, oh she’s got nurse clinic ‘cos she’s overweight.’ Interview 16

Some felt a phone-app could be effective to teach about healthy lifestyles and commented on the benefits of having ‘instant access’ information. However, they explained that in addition to phone app ‘generic information’, individualised support would be needed via a different route (eg, face to face or telephone).

### Multi-professional approach with trained, experienced staff

Some parents saw benefits in meeting a mix of professionals, including dietitians, psychologists and youth workers (table 3). The role of the psychologist was observed as helping to understand the connection between foods and emotions; whereas, the dietitian was often seen as a more ‘factual’ information gathering and providing role. A few parents commented that the most important thing is that intervention staff are trained and experienced in dealing with children’s weight management.

### Change takes time

When discussing the desired frequency of contact within the context of an intervention, many parents felt that sessions should be fortnightly or monthly rather than weekly (table 3). Some parents commented that change takes time and weekly intervals do not allow sufficient time to contemplate and effect changes. A few parents though felt weekly then reducing frequency to fortnightly or monthly was appropriate.

### Weight monitoring acceptable if done in the ‘right way’ and with privacy

Most parents were happy for weight monitoring during the intervention to help their child reach a healthy weight, providing it was done in the ‘right way’, with the focus of discussions being on health rather than weight (table 3). The importance of privacy of weight measurements, avoiding ‘singling out’ a child, was made clear.

### Interest in a parent peer support group

The telephone interviews explored the potential for a parent-peer support group (table 4). Most felt this would be beneficial, with some describing positive experiences from other peer support groups (eg, autism). A minority were less keen, due to a lack of time (work/family pressures), or discomfort discussing issues in a group setting.

**Table 4:** Parent-peer support group (Source: telephone interview data, n=18)

Theme	Quotes
Benefits of a parent-peer support group	‘I think it would be really nice and sometimes if… even if its your child that’s overweight and not you, you feel sort of almost embarrassed really and so somebody else… for other parents to be in a similar position I think would be really nice.’ Interview 12
Positive experience of peer support in other settings	‘Certainly looking at the parent support groups that I’ve been involved in with autism and attachment and everything, they’re what’s kept me sane so yes I think any support from people that are going through the same thing, having the same issues that you’re having or even slightly different issues but around the same basic thing is always helpful.’ Interview 10
Need for a group facilitator	‘And I think that’s how it becomes confusing in like everybody’s got a difference of opinion and you get a bit of conflicting advice and I think that’s what’s put me in the position….I’m hooked on too many forums and I’ve read too much and you start getting a bit confused as to what’s the right… So I think having that as a support network is good but get someone facilitating it and giving the research-led information, not just parents’ opinions because as you know all parents have got different points, not necessarily based on what research has shown or what is right’. Interview 5
Importance of a safe space	‘I wouldn’t perhaps ask advice from other people unless I knew that it was like an okay place to talk about it without any judgement.’ Interview 14

Some wanted a group facilitator to manage differing opinions between parents and formulate the ‘right’ advice. A few emphasised that for any parent-peer support group, it was important the group felt safe in a confidential, non-judgemental space.

## Discussion

### Main findings

Over three-quarters of parents worried about their child’s weight wanted support to help their child reach a healthier weight. Nearly all parents were happy for the adult WMP to signpost to this support. Around half of parents (47%) not worried about their child’s weight were still interested in a height and weight check, to assess their child’s health status.

The data highlights that there is ‘no one size fits all’, in terms of parents’ preferred intervention format (parent-led vs family-based) and delivery method, with different approaches suiting different individuals. Participants recognised the benefits of a multiprofessional approach and commented that change takes time, often preferring fortnightly or monthly sessions, compared with weekly. Child weight monitoring during an intervention was acceptable to most parents, if this was handled in the ‘right way’. Many parents felt that a parent-peer support group would be a useful opportunity to support each other.

### Contextualisation and implications of findings

There is considerable emphasis on ‘family-based’ child weight management services,^17^ but far less emphasis in all adult weight management tiers to directly address the needs of children within the family, who might benefit from support. This is evident within the NICE child lifestyle weight management guidance (CG189), which includes a recommendation to ‘encourage parents of children and young people who are living with overweight or obesity to lose weight if they are also living with overweight or obesity’.^14^ However, there is no such recommendation in equivalent adult lifestyle weight management guidance (PH53), to signpost parents worried about their child’s adiposity, to child weight management support.^18^ NICE acknowledge that lack of awareness about child weight management services is a barrier to children and their families accessing support.^8^ However, their recommendations regarding raising awareness of child lifestyle weight management programmes (NQS 94, QS5)^19^ do not include any advice to signpost parents attending adult weight management programmes, such as Slimming World or WW (formerly Weight Watchers), to childhood support services.

This mis-matched approach to the entire family, between the child and adult weight management guidance, represents a missed opportunity to help support families reach a healthy weight together. In our study, nearly all parents who were interested in childhood weight management support were happy for the adult WMP to signpost them to this support. Furthermore, these families are potentially in the optimal position to support their child reach a healthier weight given the potential collateral effects observed when a parent addresses their own weight.^12 20 21^

Parents often find it difficult to recognise and accept when their child is overweight.^22^ Interestingly, nearly half of parents who were not concerned about their child’s weight would still like to have a weight status check, despite the NCMP in England, offering weight status measurements in reception and year 6.^23^ Annual child weight monitoring and feedback programmes show potential benefit to support healthier child growth trajectories.^24^ Furthermore, the receptiveness of parents to a weight measurement indicates that it may be possible to reach more families with the offer of child weight management support, where the subjective assessment of parents (ie, normal weight) differs to the objective independent assessment (ie, overweight).

Parental views on intervention format concord with current recommendations, recognising the need for individually tailored interventions with a multiprofessional approach^17^ and outcome measures including weight measurement.^25^ However, parents did express that a weekly interval for the delivery of an intervention may be too frequent, which is at odds with the ‘12-week, weekly session’ format^26^ of many commissioned interventions for children, and warrants further research, especially given the very limited literature supporting this format efficacy.^27^

Social, peer parental support has been identified as a factor supporting efficacy of child weight management interventions^28^ and recognised by parents to be of value within the context of an intervention.^29^ This study supports the potential value of a parent-peer support group warranting further exploration, independent to a traditional child weight management intervention.

### Strengths and weaknesses of the study

Survey and telephone interview participants were not known to the researcher prior to taking part, and participants were not explicitly informed that the researcher was also a general practitioner, to reduce response bias. However, the method of recruitment mainly via the Slimming World website may have led to selection bias, by limiting representation from underserved communities. Most participants were female; this reflects the predominantly female membership of Slimming World.^30^ It was not possible to ascertain the survey response rate, as we do not know how many parents of children aged 5–11 years viewed the recruitment advert, or were directed to the survey link by Slimming World group Consultants.

The children were not measured; however, parents usually underestimate weight; hence, it is unlikely parents would express concern for a child with a ‘healthy’ weight, to have overweight or obesity.^22^ As interviews were conducted by phone, others may have been present at the participant’s location, and influenced the answers provided. The study was designed to include only adult parent participants, but future studies should explore children’s perspectives.

## Conclusion

This study suggests a potential novel referral pathway into child weight management programmes, through signposting parents attending adult weight management programmes. Support for families to help their child reach a healthy weight should be tailored to the individual, with intervention design recognising that change takes time and piloting parent-peer support groups as an adjunct. Future research should pilot this novel referral pathway and explore outcomes such as recruitment and retention, compared with traditional referral pathways.

## Data Availability

Data are available upon reasonable request.
