# Supplementary material for: Feasibility of engaging parents attending an adult weight management programme with child weight management support: a mixed methods study
Source: BMJ Paediatr Open. 2025 Jan 17;9(1):e002975. doi: 10.1136/bmjpo-2024-002975 (PMC11749568; doi:10.1136/bmjpo-2024-002975)
Supplement: Supplementary file 1 [file bmjpo-2024-002975.reviewer_comments.pdf]

PEER REVIEW HISTORY

BMJ Paediatrics Open publishes all reviews undertaken for accepted manuscripts. Reviewers are asked to complete a checklist review form and are provided with free text boxes to elaborate on their assessment. These free text comments are reproduced below.

ARTICLE DETAILS

**TITLE (PROVISIONAL)**	Feasibility of engaging parents attending an adult weight management programme with child weight management support: a mixed methods study.
**AUTHORS**	Mears, Ruth; Searle, Aidan; sharp, deborah; Jago, Russell; Shield, Julian

VERSION 1 - REVIEW

**REVIEWER NAME**	***Jenny Sohn***
**REVIEWER AFFILIATION**	Sydney Children's Hospital Randwick
**REVIEWER CONFLICT OF INTEREST**	Not applicable
**DATE REVIEW RETURNED**	15-Nov-2024

**GENERAL COMMENTS**	This is an interesting, well-written article with good qualitative data regarding perspective of parents with weight/obesity issues.<br><br>It would be better to reference Slimming World as adult weight management program (WMP), as initially described rather than repeating the commercial company name repeatedly throughout the article. (E.g. Page 4: Adults with overweight or obesity may seek help to reach a healthier weight by joining Slimming World, Page 5: …willing to be signposted by Slimming World to support for their child.)

VERSION 1 – AUTHOR RESPONSE

Reviewer: 1

Comment 1: This is an interesting, well-written article with good qualitative data regarding perspective of parents with weight/obesity issues.

Response 1: Thank you for taking the time to review this article and your comments.

Comment 2: It would be better to reference Slimming World as adult weight management program (WMP), as initially described rather than repeating the commercial company name repeatedly throughout the article. (E.g. Page 4: Adults with overweight or obesity may seek help to reach a healthier weight by joining Slimming World, Page 5: …willing to be signposted by Slimming World to support for their child)

Response 2: Thank you for your comments. We have adjusted the title to remove reference to Slimming World, instead referring to 'an adult weight management programme.' We have also changed the abstract 'results' and 'conclusion' section, to refer to 'adult WMP' rather than 'Slimming World'. We have adjusted the wording in the 'What this study adds' section. In the introduction, we have given some information about Slimming World, so that the reader has an understanding of the

nature of the adult weight management programme involved in this study. At the end of the 'introduction' section in the aims, we now refer to adult WPM rather than SW. Similarly, we have changed terminology on page 8, 11 and 12 and in Figure 1.

VERSION 2 – REVIEW

**REVIEWER NAME**	***Santuri Rungan***
**REVIEWER AFFILIATION**	Croydon Community Health Centre, Community Paediatrics
**REVIEWER CONFLICT OF INTEREST**	n/a
**DATE REVIEW RETURNED**	12-Dec-2024

**GENERAL COMMENTS**	Thank you for this revision, the article will now be accepted for publication.
